# Robust, high-productivity phototrophic carbon capture at high pH and alkalinity using natural microbial communities

**DOI:** 10.1186/s13068-017-0769-1

**Published:** 2017-03-29

**Authors:** Christine E. Sharp, Sydney Urschel, Xiaoli Dong, Allyson L. Brady, Greg F. Slater, Marc Strous

**Affiliations:** 1 Department of Geosciences, University of Calgary, 2500 University Drive NW, EEEL 509, Calgary, AB T2N 1N4 Canada; 2 School of Geography and Earth Science, McMaster University, Hamilton, ON Canada

**Keywords:** Algae biofuel, *Phormidium kuetzingianum*, Mixed-community, Microbial ecology, Biofilm

## Abstract

**Background:**

Bioenergy with carbon capture and storage (BECCS) has come to be seen as one of the most viable technologies to provide the negative carbon dioxide emissions needed to constrain global temperatures. In practice, algal biotechnology is the only form of BECCS that could be realized at scale without compromising food production. Current axenic algae cultivation systems lack robustness, are expensive and generally have marginal energy returns.

**Results:**

Here it is shown that microbial communities sampled from alkaline soda lakes, grown as biofilms at high pH (up to 10) and high alkalinity (up to 0.5 kmol m^−3^ NaHCO_3_ and NaCO_3_) display excellent (>1.0 kg m^−3^ day^−1^) and robust (>80 days) biomass productivity, at low projected overall costs. The most productive biofilms contained >100 different species and were dominated by a cyanobacterium closely related to *Phormidium kuetzingianum* (>60%).

**Conclusion:**

Frequent harvesting and red light were the key factors that governed the assembly of a stable and productive microbial community.

**Electronic supplementary material:**

The online version of this article (doi:10.1186/s13068-017-0769-1) contains supplementary material, which is available to authorized users.

## Background

Assessments by the International Panel on Climate Change (IPCC) have concluded that to limit increases in mean global surface temperature to below 2 °C [1], net human-driven carbon emissions must be eliminated by the end of this century. One of the most promising carbon mitigation strategies is bioenergy with carbon capture and storage (BECCS) [2]. Current BECCS strategies involve the growing of trees and crops that extract carbon dioxide (CO_2_) from the atmosphere, burning them in power plants, stripping the resultant CO_2_ from the waste gas and storage via CO_2_ injection into geological formations [3]. However, implementing BECCS at the required scale would have vast ecological consequences, risk inflating food prices and create a carbon debt [4].

Alternatively, one could generate biomass using phototrophic microorganisms such as algae or cyanobacteria. Compared to the agricultural production of more ‘traditional’ fuel crops such as corn, cultivation of phototrophic microorganisms has much higher projected yields per acre and can be constructed on degraded or otherwise unproductive land [5]. However, the use of phototrophic microorganisms for carbon capture and biofuel production is hindered by poor economic feasibility. It remains difficult to design a system in which energy is actually produced rather than consumed [6, 7]. Operational costs are high due to the energy requirements of bubbling gas containing carbon dioxide through the open pond or photobioreactor. Studies haves shown that the cost of carbon dioxide supply can constitute approximately 50% of the cost of biomass production in raceway pond systems [8]. The use of high pH and alkalinity can improve transfer of CO_2_ into the culture medium and uncouples CO_2_ absorption and biological uptake, leading to lower energy requirements and costs [9–11]. Haloalkaliphilic eukaryotic algae and cyanobacteria can use bicarbonate instead of CO_2_ at high pH [12, 13]. Thus, CO_2_ could be transferred to the growth medium in a separate trickling filter with low pressure drop and driven by the pH equilibrium, reducing energy requirements and operational costs of the carbon supply [9, 10]. The growth of algae using bicarbonate as a method to sequester carbon dioxide has previously been suggested [9–11]. Growth in biofilms reduces energy and costs of biomass growth and harvesting as compared to suspended cultivation due to the lower cultivation volume and higher biomass densities [14, 15]. To harvest algal biomass from a suspended culture, the culture is commonly concentrated via sedimentation or flocculation to around 1% (dry basis) then further concentration using centrifugation to 20% (dry basis) [16, 17]. These processes are energy intensive. In most algal biofilm systems, the harvested biomass has a solid content of 10–20% (dry basis); this is similar to that of the post-centrifuge suspended biomass [18, 19].

Apart from high costs, algal biotechnology also struggles with a lack of robustness. Present approaches typically focus on the axenic cultivation of a single strain, such as *Spirulina*, *Nannochloropsis*, *Chlorella* or *Dunaliella*. However, at large scale, aseptic conditions are difficult (and costly) to maintain [20]. Ecological processes such as invasion by other algae species, decimation by grazers, fungi and/or viral infection lead to process instability [21, 22]. Indeed, some systems have seen the complete destruction of the microalgae crops within 48 h of protozoan detection [23]. Most algal biotechnology research is focused on tackling this problem from a genetic or cellular perspective and the usage of polycultures of pure strains of microalgae [24]. Alternatively, robustness could also be improved from an ecological perspective, by making use of mixed microbial communities consisting of both microalgae and bacteria instead of axenic strains. One of the basic principles of microbial ecology is that species diversity promotes ecosystem productivity and stability [25]. This principle also holds true for engineered ecosystems such as traditional wastewater treatment plants. These plants are robust and inherently open systems that rely on dozens (or more) species of different microorganisms coming together to transform the waste into less harmful substances [26].

In this study, an ecological approach was applied to realize a stable phototrophic carbon capture system. In this system, mixed-community biofilms were cultivated at high pH and high alkalinity in phototrophic bioreactors. The analysis included (i) characterization of the microbial community structure by high-throughput 16S rRNA gene sequencing, (ii) biofilm productivity measured as ash-free volumetric and surface areal productivity, and (iii) biofilm productivity measured as oxygen production.

## Results

### Initial phototrophic cultivation

**Fig. 1:**
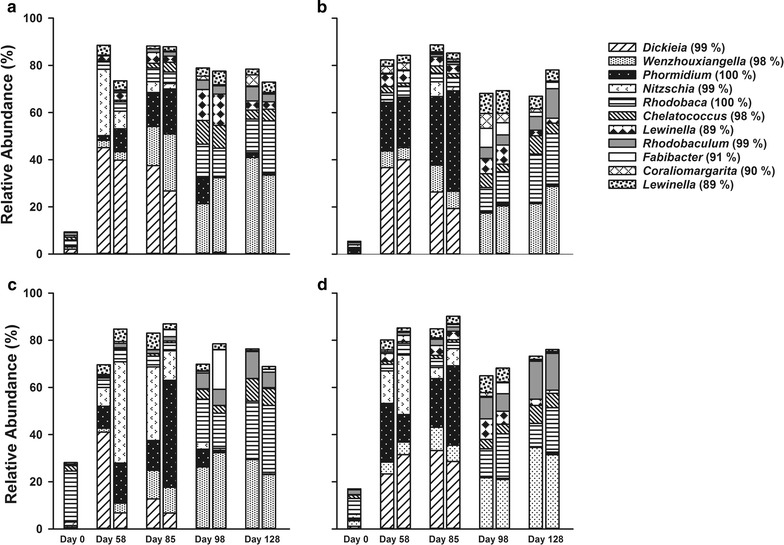
Photobioreactor microbial community composition based on 16S rRNA gene amplicon sequencing. The figure shows relative abundance and taxonomic assignments of the most abundant populations (>2% per lake average abundance) for duplicate photobioreactors inoculated with **a** Deer Lake, **b** Goodenough Lake, **c** Last Chance Lake and **d** Probe Lake mats at 0, 58, 85, 98 and 128 days. Eukaryotic algae sequences are based on BLAST identification of sequence reads identified as chloroplasts

Duplicate flat panel laboratory bioreactors, fed with a high pH, high alkalinity medium (pH 10, 0.5 mol L^−1^ dissolved carbonate), were inoculated individually with microbial mats from four soda lakes located in British Columbia, Canada [Last Chance Lake (LCL-M), Probe Lake (PL-M), Deer Lake (DL-M) and Goodenough Lake (GEL-M)]. 16S and 18S rRNA gene sequencing showed that these microbial mats harboured a diverse microbial community. The biofilms were harvested at four different time points (day 0, 58, 85, 98 and 128). Few populations from the initial microbial community are shown in Fig. 1 as the photobioreactor selected populations were not initially abundant. Bioreactors were initially grown under ambient light conditions (15 µmol photon m^−2^ day^−1^). After harvesting on day 85 the bioreactors were artificially illumined with full spectrum grow lights at an intensity of 65 µmol photon m^−2^ day^−1^ with a 16:8 h light:dark photoperiod. Bacterial 16S ribosomal RNA amplicon libraries showed that biofilms cultivated from each of the four lakes were initially (days 58 and 85) dominated by a mixture of three populations related to the cyanobacterium *Phormidium kuetzingianum* (identity 100%), and the bacillariophyta (diatoms) *Nitzschia thermalis* (identity 99%, based on the chloroplast 16S ribosomal gene) and *Dickieia ulvacea* (identity 99% based on the chloroplast 16S ribosomal gene) (Fig. 1; Additional file 1: Table S1, Additional file 2: Table S2, Additional file 3: Table S3, Additional file 4: Table S4). In all photobioreactors from the four soda lakes, a dramatic shift in microbial community structure was observed between days 85 and 98. This shift resulted in an almost total loss of the oxygenic phototrophs *P. kuetzingianum, N. thermalis* and *D. Ulvacea,* concurrent with increases in the relative abundances of populations related to the *Proteobacteria* genera *Rhodobaca* (identity 100%), *Rhodobaculum* (identity 99%), *Wenzhouxiangella* (identity 98%) and *Chelatococcus* (identity 98%); the *Bacteroidetes* genera *Lewinella* (two groups, identity 89%) and *Fabibacter* (identity 91%), and the *Verrucomicrobia* genus *Coraliomargarita* (identity 90%) (Fig. 1; Additional file 1: Table S1, Additional file 2: Table S2, Additional file 3: Table S3, Additional file 4: Table S4). This shift in microbial community composition was also apparent from a nonmetric multidimensional scaling (NMDS) plot based on the Bray–Curtis similarity index (Additional file 5: Figure S1). Two distinct clusters were observed, samples from days 58 and 85 in one cluster, and days 98 and 128 in the other. Formation of these clusters was independent of the source of the inoculum, even though the soda lake mat samples harboured very different microbial communities.

### Selection of phototrophic community using wavelength of light

**Fig. 2:**
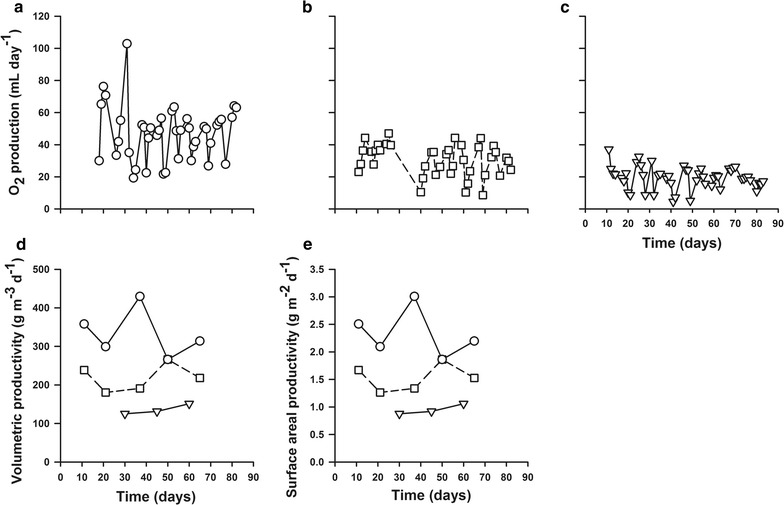
Productivity of mixed-community phototrophic biofilms grown at pH 10, 0.5 M carbonates under red (**a**, *open circle*), white (**b**, *open square*) and blue (**c**, *inverted triangle*) light shown as oxygen production, volumetric (**d**) and surface areal (**e**) productivities

A second set of flat panel photobioreactors consisted of three sets of replicated thin rectangular plastic vessels with an illuminated surface area of 197.5 cm^2^, ground area of 18 cm^2^ and an inner volume of 138.25 cm^3^. The geometry of these flat panel bioreactors was designed to provide a more even distribution of the medium flow. Each set was illuminated with a different wavelength of light; full spectrum (white), 590–656 nm (red), or 422–496 nm (blue) at a light intensity of 80 µmol photon m^−2^ day^−1^ with a 16:8 h light: dark cycle to select for growth of either the cyanobacteria or diatoms. Each photobioreactor was inoculated with a mixture (equal weight, w/w) of microbial mats from the four soda lakes and supplied with the same alkaline medium (pH 10, 0.5 mol L^−1^ dissolved bicarbonate). The productivity was estimated by two independent methods: quantification of produced oxygen by real-time mass spectrometry and quantification of ash-free dry weight after each biomass harvest. The biomass was harvested whenever a decline in oxygen production was observed, typically >5 mL day^−1^ (Fig. 2a–c). The walls of these photobioreactors contained thin grooves, which enabled part of the bacteria to evade harvesting, leading to effective regrowth after each harvest.

All photobioreactors showed robust productivity for the entire 82-day experiment. Bioreactor productivity was evaluated as (i) volumetric productivity, in terms of ash-free dry weight biomass produced per unit reactor volume per unit time (expressed as g m^−3^ day^−1^) and (ii) surface area productivity, in terms of ash-free dry weight biomass produced per unit of reactor illuminated surface area per unit time (expressed as g m^−2^ day^−1^). Volumetric and surface area productivity was the highest for biofilms grown under red light, up to a maximum of 429 g m^−3^ day^−1^ (average 333 g m^−3^ day^−1^), and 3.0 g m^−2^ day^−1^ (average 2.3 g m^−2^ day^−1^), respectively and were stable over time (Fig. 2d, e). The white-light photobioreactor had a maximum volumetric productivity of 266 g m^−3^ day^−1^ (average 218 g m^−3^ day^−1^) and a maximum surface areal productivity of 1.9 g m^−2^ day^−1^ (average 1.5 g m^−2^ day^−1^) (Fig. 2d, e). The blue-light photobioreactor displayed the lowest productivities with a maximum volumetric productivity of 151 g m^−3^ day^−1^ (average 136 g m^−3^ day^−1^) and a maximum surface areal productivity of 1.1 g m^−2^ day^−1^ (average 0.95 g m^−2^ day^−1^) (Fig. 2d, e).

Oxygen production rates were in good agreement with the biomass productivity estimated from biomass ash-free dry weights with the red-light photobioreactor consistently having the highest O_2_ production (experiment average 46 mL day^−1^). The white-light photobioreactor had an average O_2_ production rate of 26 mL day^−1^, while the blue-light photobioreactor was on average 19 mL day^−1^ (Fig. 2a–c).

**Fig. 3:**
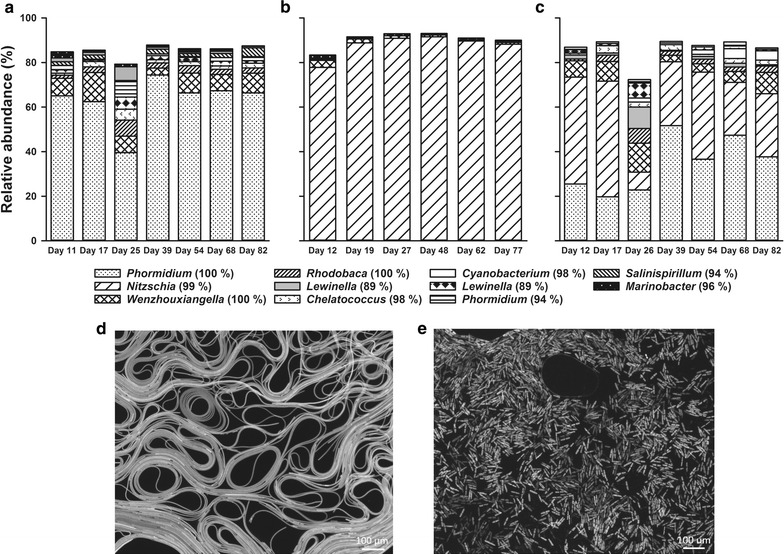
Photobioreactor microbial community composition based on 16S rRNA gene amplicon sequencing and fluorescence microscopy. The figure shows relative abundance and taxonomic assignments of the most abundant populations (>1% relative abundance) for photobioreactors illuminated with red (**a**), blue (**b**), and white (**c**) light, during 77 days. Fluorescence microscopy images of biomass from the red light (**d**) showing the dominance of a cyanobacterium closely related to *Phormidium* and blue light (**e**) showing the dominance of a diatom closely related to *Nitzschia*. Eukaryotic algae sequences are based on BLAST identification of sequence reads identified as chloroplasts

To identify changes in the microbial community structure over time, total DNA was isolated from the biofilms at seven time points (six for blue light) and bacterial 16S rRNA gene amplicon libraries were generated. Biofilms grown under red light were dominated (>60%) by a population related to the cyanobacterium *P. kuetzingianum* (identity 100%, Additional file 6: Table S5), contained at least eight different species at >1% average relative abundance (Fig. 3a) and over 150 different species at <1% relative average abundance. The blue-light microbial community was dominated (>80%) by a population related to the bacillariophyta (diatom) *N. thermalis* (identity 99% based on the chloroplast 16S ribosomal gene, Additional file 6: Table S5). This biofilm contained two other species at >1% average relative abundance (Fig. 3b; Additional file 6: Table S5) and over 150 different species at <1% relative average abundance. Fluorescence microscopy images of biomass from the red-light photobioreactor (Fig. 3d) showed that the cyanobacterium *P. kuetzingianum* dominated these microbial communities, whereas the blue-light photobioreactors (Fig. 3e) were dominated by the diatom *N. thermalis*. Biofilms from the white-light photobioreactors contained a mixture of *P. kuetzingianum* and *N. thermalis,* observed in the red and blue bioreactors, respectively (Fig. 3c), and >150 different species in total. A NMDS plot based on a matrix of Bray–Curtis community similarities identified a broad separation according to wavelength of light (Additional file 7: Figure S2). Similar to the first experiment, a distinct separation was observed between the inoculum/soda lake microbial mats and the wavelength-selected bioreactor microbial communities. The community compositions were in good agreement with the visual appearance of the photobioreactors; the red-light biofilms were green, the blue-light biofilms were brown and the white-light biofilms contained a mixture of (spatially separated) green and brown zones.

The harvested biomass from the red-light photobioreactor had more favourable settling properties than the biomass from the blue-light photobioreactor. After harvesting, the biofilms from the blue-light photobioreactor rapidly dispersed, yielding mainly single cells. Fragments produced from the biofilms of the red-light photobioreactor during harvesting rapidly coagulated, forming a large, single globule of biomass that settled almost immediately and facilitated harvesting.

### Optimization of cultivation

**Fig. 4:**
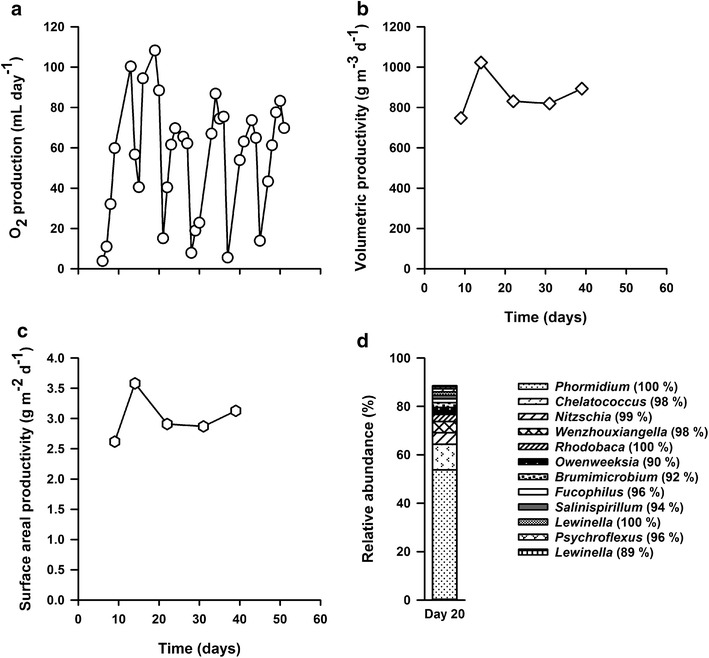
Productivity of a mixed-community biofilm grown at pH 9.0, 0.5 M bicarbonate illuminated with red light as a function of oxygen production (**a**) volumetric (**b**) and surface areal (**c**) productivity. **d** Relative abundance and taxonomic affiliations of abundant populations (>1% relative abundance) based on 16S rRNA gene sequencing on day 20. Eukaryotic algae sequences are based on BLAST identification of sequence reads identified as chloroplasts

Based on the productivity data and ease of harvesting of the red-light photobioreactors, this condition was chosen for further optimization. To improve the volumetric productivity, the flat panel photobioreactors were redesigned to have an illuminated surface area of 197.5 cm^2^ and inner volumes of 34.6 or 69.1 cm^3^ [from here-on referred to as the high-productivity (HP) bioreactor]. These bioreactors were fed with a high pH, high alkalinity medium (pH 9, 0.5 mol L^−1^ dissolved bicarbonate), inoculated with a mixture of microbial mats from the four different soda lakes and received 80 µmol photon m^−2^ day^−1^ of red light in a 16:8 illumination cycle. The biomass grown in the 34.6 cm^3^ volume bioreactor did not grow efficiently as oxygen bubbles became stuck in the small depth of liquid resulting in a poorly formed biofilm and therefore will not be discussed further. Oxygen production for the HP bioreactor was stable over time and was similar if not slightly higher than the O_2_ production of the previous red-light bioreactor (Fig. 4a), average 55 mL day^−1^ versus average 46 mL day^−1^. This is likely a result of the lower pH of this system (pH 9 versus 10). Volumetric productivities were higher than those achieved in the previous experiments, up to 1022 g m^−3^ day^−1^, average 862.91 g m^−3^ day^−1^ (Fig. 4b) as compared to the 333.3 g m^−3^ day^−1^ on average previously observed (Fig. 2d). Surface areal productivities were also slightly higher, average 3.0 g m^−2^ day^−1^ (Fig. 4c), as compared to 2.3 g m^−3^ day^−1^ average for the red-light photobioreactor (Fig. 2e).

The microbial community on day 20 was dominated (>50%) by a bacterium closely related to the cyanobacterium *P. kuetzingianum* (identity 100%, Fig. 4d; Additional file 8: Table S6) and contained at least 11 other species at >1% relative average abundance. Interestingly, despite illumination with red light, this microbial community still contained a small amount (<5% relative abundance) of a bacillariophyta closely related to *N. thermalis* (identity 99% based on the chloroplast, Fig. 4d; Additional file 8: Table S6). The presence of *N. thermalis* did not appear to affect the overall productivity of the system as both the oxygen production and volumetric productivities were stable over time and remained higher than the red pH 10 bioreactor. A NMDS plot based on a matrix of Bray-Curtis community similarities showed that the HP bioreactor day 20 microbial community clustered within the red wavelength bioreactors from the previous experiment (Additional file 7: Figure S2), indicating that ecological selection was reproducible.

The total lipids content of the biomass on day 20 was 13.4% dwt (of the total biomass dry weight). Volumetric lipid productivity was calculated as the product of lipid content and biomass productivity. The lipid productivity of the biomass on day 20 was calculated to be 115.4 mg lipids L^−1^ day^−1^. The fatty acid concentration was 17.6 mg g^−1^ of total lipid for the day 20 HP bioreactor biomass. The fatty acid profile is shown in Additional file 9: Table S7. The two most dominant fatty acids were C16:1 (36.7%, multiple isomers) and Palmitic acid (C16:0, 26.7%), with smaller proportions of C16:2 (10.3%), C20:5 (8.0%) and C18:1 (5.8%). Overall, the biomass fatty acids were 34.4% saturated, 42.9% monounsaturated (MUFA) and 22.8% polyunsaturated (PUFA).

## Discussion

The sampled alkaline soda lakes each harboured a completely different microbial community, as shown by nonmetric multidimensional scaling analysis. The same analysis showed that during photobioreactor cultivation, these communities became very similar to each other. This suggests that photobioreactor cultivation induced strong and reproducible ecological selection. In all photobioreactors from all four soda lakes, an increase in illumination intensity led to a dramatic shift in microbial community structure between days 85 and 98. This shift might have been caused by more prolific biofilm growth, leading to anoxic conditions, especially during dark periods when no oxygen was produced. These conditions selected for anoxygenic phototrophs at the expense of oxygenic phototrophs. For this reason, a second set of experiments were performed, where the biofilms were regularly harvested whenever a decrease in oxygen production was observed. To prevent the emergence of anoxic pockets during growth, the geometry of the flat panel photobioreactors was changed to create a more even distribution of the fresh medium, which might have prevented the occurrence of anoxia during dark periods. All the bioreactors in this second experiment showed long-term, robust productivity over the 82-day period. In this regard, a microbial community might outperform an axenically cultivated single strain of algae, which can show a loss of productivity caused by contamination [27, 28]. The use of high alkalinity and high pH likely also limited the contamination.

By providing only red or blue light, it was attempted to selectively grow either cyanobacteria or diatoms. Indeed, the biofilms grown under red light were dominated (>60%) by a population related to the cyanobacterium *P. kuetzingianum*, while the blue biofilms were dominated by a population related to the diatom *N. thermalis.* White-light biofilms contained a mixture of these two populations. The phototrophs serve as the sole or primary autotroph, suppling oxygen and photosynthetically derived carbon to the heterotrophic community, while the heterotrophs might promote the growth of phototrophs by proving key metabolites and scavenging wastes [29, 30]. The microbial communities in all three bioreactors were stable over time with one minor community disturbance at day 25 (red) and day 26 (white) observed. The red-light biomass was superior to both the blue-light and white-light photobioreactor biomass both in terms of productivity and ease of harvesting. Volumetric productivities of the red-light photobioreactor were a maximum of 429 g m^−3^ day^−1^, and were stable over the course of the 82 day experiment. The red-light biomass formed a thin biofilm on the walls of the photobioreactor. This biofilm was easily removed from the walls into the liquid medium via minor agitation or a low-pressure air flow. When the liquid medium was removed from the photobioreactor, the biomass easily flowed with it. The harvested biomass rapidly coagulated in a flask forming a single globule of biomass. This biomass could easily be separated from the bulk suspension by physical removal with a pipette tip. To what extent these harvesting approaches might work at large scale is a topic for further research.

Despite the large body of work showing a diversity–productivity relationship, the practical implication of using algal species diversity for enhancing productivity or stability of biotechnological systems remains largely untested. It has only been explored on a small scale in relatively few studies, using either constructed algal assemblages with ten or fewer species [31, 32] or natural assemblages with a species richness of at most 19 [33]. The evidence of a polyculture approach is promising so far. For example, Corcoran and Boeing [32] showed that the most species-rich polycultures (*n* = 6) were stable and recovered from short-term negative grazing effects by rotifers, whereas the monocultures experienced persistent negative effects. In the present study, the community disturbance at days 25–26 did not cause a corresponding decrease in oxygen production and the dominant phototrophic microbial community had re-established itself by day 39 without any human intervention (other than the usual harvesting cycling).

The microbial community from the HP bioreactor was similar to the previous red-light bioreactor, except for the presence of a small amount of a diatom related to *N. thermalis.* The HP bioreactor also displayed the highest volumetric productivities of the photobioreactors tested, up to 1022 g m^−3^ day^−1^, average 862.91 g m^−3^ day^−1^ at 80 µmol photon m^−2^ day^−1^ of red light in a 16:8 light and dark cycle. These productivities are on par with most reported previous findings for axenic cultures. Nascimento et al. [34] showed a volumetric productivity of 730 g m^−3^ day^−1^ for an axenic culture of *Chlorella vulgaris* grown in a 12:12 light and dark cycle under 140 µmol photons m^−2^ day^−1^. All other strains tested in that survey showed biomass productivities between 200 and 340 g m^−3^ day^−1^. Volumetric productivities of up to 1900 g m^−3^ day^−1^ have been observed for suspended cultures of *Phaeodactylum tricornutum* in an airlift tubular bioreactor [35]. 1470 g m^−3^ day^−1^ was measured for *Chlorella sorokiniana* cultivated as a suspended culture in an inclined tubular bioreactor [36] and 420 m^−3^ day^−1^ was measured for *Spirulina platensis* in an airlift tubular system [37].

The surface areal productivity of the HP bioreactor was also typical of a biofilm photobioreactor (maximum 3.6 g m^−2^ day^−1^). Various types of algal biofilm systems have been developed. For example, growth of *P. tricornutum* on the twin-layer biofilm photobioreactor achieved a surface biomass productivity of 1.8 g m^−2^ day^−1^ [38]. Boelee et al. [39] designed a flow-lane biofilm bioreactor and achieved areal production rates between 2.7 and 4.5 g m^−2^ day^−1^. Interestingly, their system was inoculated with municipal wastewater and the subsequent biofilm was dominated by the cyanobacterium *Phormidium autumnale*. The rotating algal biofilm cultivation system (RAB) inoculated with *C. vulgaris* has demonstrated productivities of 3.51 g m^−2^ day^−1^ (laboratory scale) [18] and 5.80 g m^−2^ day^−1^ (pilot scale) [40, 41]. Schnurr et al. [42] cultivated *Nitzschia palea* and *Scenedesmus obliquus* biofilms on a flat plate parallel horizontal photobioreactor with areal productivities of 2.8 and 2.1 g m^−2^ day^−1^. These results suggest that the cultivation of a mixed-community microbial biofilm at high pH and alkalinity results in a system that is both stable and highly productive compared to common axenic culture systems.

The total lipid content of the HP bioreactor biomass on day 20 was 13.4% dwt. This value is consistent with reported literature values for other Cyanobacteria such as *Spirulina* (7–13% dwt) and *Oscillatoria* (7% dwt) grown under nutrient-replete conditions [43]. Interestingly, the total lipid content of some freshwater *Phormidium* and *Oscillatoria* sp. is substantially higher when directly sampled from the environment, 26.7 and 28.1%, respectively [44]. The highest lipid yields under nutrient-replete cultivation conditions are typically seen for the *Chlorophyta* (green algae), 13–31% dwt, and the *Bacillariophyceae* (diatoms), 21–51% dwt [34, 43]. The lipid productivity of the biomass on day 20 was calculated to be 115.4 mg L^−1^ day^−1^. This value is remarkably high compared to other axenic culture systems and is a product of the very high volumetric productivity and the average cyanobacterial lipid content of this microbial biomass. Few species of microalgae have been shown to have lipid productivities above 115 mg L^−1^ day^−1^: *C. vulgaris* (204.91 mg L^−1^ day^−1^) [34], *Ettlia oleoabundans* (164 mg L^−1^ day^−1^) [43] and *Amphora* (160 mg L^−1^ day^−1^) [43]. The dominance of the C_16_ fatty acid is common amongst cultured *Phormidium* species [45]. Overall, the biomass lipids were 34.4% saturated, 42.9% monounsaturated (MUFA) and 22.8% polyunsaturated (PUFA). As opposed to fuels composed of PUFAs, the high proportion of saturated and MUFAs (combined 77.2%) may incur fewer problems with fuel polymerization during combustion [46].

## Conclusion

In this study, the cultivation of a mixed-community microbial biofilm at high pH and alkalinity is shown to comprise a productive and stable phototrophic carbon capture system. High volumetric productivities were observed for a mixed microbial community dominated by a cyanobacterium closely related to *P. kuetzingianum* (maximum 1022 g m^−3^ day^−1^, average 862.91 g m^−3^ day^−1^) grown at pH 9, 0.5 mol L^−1^ total carbonates and 80 µmol photon m^−2^ day^−1^. This high productivity combined with the predicted fourfold lower cost of a high pH, high alkalinity system [9] has the potential to reduce the overall cost of phototrophic carbon capture systems. Fatty acid content and profiles were consistent with previously reported axenic cultures of cyanobacteria. It should be noted that further cost reductions will still be needed to make production of biofuels from algae economically feasible, and there is no consensus on the best method of growing and harvesting algal biomass. Furthermore, production of higher added-value products such as pharmaceuticals or animal feedstock might not be possible with mixed microbial communities. The present study is an excellent starting point to investigate additional improvements to further reduce the costs of CO_2_ conversion with algae.

## Methods

### Study site

Microbial mat samples were collected in June 2014 and May 2015. Based on previous data [47, 48], benthic microbial mats from four soda lakes located on the Cariboo Plateau, British Columbia were chosen: Last Chance Lake (LCL-M), Probe Lake (PL-M), Deer Lake (DL-M) and Goodenough Lake (GEL-M). Mats were collected away from the shore (PL-M, DL-M and GEL-M) in sterile screw cap tubes. LCL-M mats were collected next to shore as they were not observed away from shore. The temperature of the four lakes ranged from 15.5 to 25.2 °C and pH from 10.2 to 10.3. Additional file 10: Table S8 provides detailed information for each lake. Mats were immediately placed at 4 °C. Collected mats from each location were pooled and incubations were set up within 10 days of sampling.

### Photobioreactor cultivation

#### Initial cultivation

Mixed-community microbial mats from each soda lake sampled in June 2014 were grown in pH 10, 0.5 mol L^−1^ dissolved carbonate media (medium 3) in 40 mL duplicate flat plate photobioreactors under continuous flow cultivation (0.14 mL min^−1^, dilution rate 4.0 day^−1^). The high pH, high alkalinity media contained (L^−1^): 39.6 g Na_2_CO_3_, 14.4 g NaHCO_3_, 6 g NaCl, 1 g K_2_HPO_4_, 246 mg MgSO_4_·7H_2_O, 218 mg NH_4_Cl, 10 mg SiO_2_ and 1 mL trace element solution. The trace element solution contained (L^−1^): 500 mg Triplex III (EDTA), 200 mg FeSO_4_·7H_2_O, 10 mg ZnSO_4_·7H_2_O, 3 mg MnCl_2_·4H_2_O, 30 mg H_3_BO_3_, 20 mg CoCl_2_·6H_2_O, 1 mg CuCl_2_·2H_2_O, 2 mg NiCl_2_·6H_2_O and 3 mg Na_2_MoO_4_·2H_2_O [49]. From day 85 onward, the flat plates were illuminated with full spectrum fluorescent lamps (SunBlaster T5HO 6400K) with a light intensity of 65 µmol photon m^−2^ day^−1^ with a 16:8 h light:dark photoperiod. Light intensity was measured using a laboratory photosynthetically active radiation (PAR) sensor (Licor Biosciences, Lincoln, NE, USA). All photobioreactors were maintained at a temperature of 21 ± 1 °C. Replicated photobioreactors were inoculated with homogenized microbial mats from each soda lake. Samples for community analysis were obtained by scraping ~95% of the biomass off the bioreactor walls, allowing for regrowth before the next time point. The composition of the mixed-community biofilms was determined using 16S rRNA gene amplicon sequencing (see “Microbial community analysis” below). Samples for DNA extraction were immediately frozen at −80 °C.

#### Wavelength selection

Mixed-culture biofilms cultivated from May 2015 soda lake microbial mats were grown in pH 10, 0.5 mol L^−1^ dissolved carbonate media (medium 3) in flat plate photobioreactors with continuous flow (0.4 mL min^−1^, dilution rate 4.0 day^−1^). The flat plate photobioreactors were thin rectangular plastic vessels with an illuminated surface area of 197.5 cm^2^, ground area of 18 cm^2^ and an inner volume of 138.25 cm^3^. A schematic of the photobioreactors is shown in Additional file 11: Figure S3. The flat plates were illuminated with fluorescent lamps (SunBlaster T5HO 6400 K) of different wavelengths; full spectrum (white) 480–580 nm (green), 590–656 nm (red), or 422–496 nm (blue) at a light intensity of 80 µmol photon m^−2^ day^−1^ with a 16:8 h light:dark photoperiod. Light intensity was measured using a laboratory PAR sensor (Licor Biosciences, Lincoln, NE, USA). All photobioreactors were maintained at a temperature of 25 ± 2 °C. Biofilm oxygen production was measured in real-time with an Agilent Mass Spectrometer MS5977A for 1 h each day. Biofilms were harvested from the photobioreactors when the oxygen production was lower than the previous day. Biomass productivity was determined as the biofilm ash-free dry weight collected during harvesting divided by the number of days between harvests. The composition of the mixed-community biofilms was determined using 16S rRNA gene amplicon sequencing (see “Microbial community analysis” below). Fluorescence microscopy images were collected based on the pigment autofluorescence of phototrophs (Zeiss, X-cite 120 LED with a Cy5 excitation filter).

#### Optimization of cultivation

Mixed-culture biofilms were grown from May 2015 soda lake samples in pH 9.0, 0.5 mol L^−1^ dissolved carbonate media (medium 3.9) in flat plate photobioreactors under continuous cultivation (0.21 mL min^−1^, dilution rate of 4.0 day^−1^). Medium 3.9 is defined as medium 3 (see above) with the pH adjusted to 9.0 with HCl. The flat plate photobioreactors were thin rectangular plastic vessels with an illuminated surface area of 197.5 cm^2^, ground area of 18 cm^2^ and an inner volume of either 34.56 or 69.13 cm^3^. The flat plates were illuminated with a red fluorescent lamp (SunBlaster T5HO 6400 K) at a light intensity of 80 µmol photon m^−2^ day^−1^ with a 16:8 h light: dark photoperiod. Light intensity was measured using a laboratory PAR sensor (Licor Biosciences, Lincoln, NE, USA). All photobioreactors were maintained at a temperature of 25 ± 2 °C. Biofilm oxygen production was measured in real-time on an Agilent Mass Spectrometer MS5977A for 1 h each day. Biofilms were harvested from the bioreactors when the oxygen production was lower than the previous day. Biomass productivity was determined as the biofilm ash-free dry weight collected during harvesting divided by the number of days between harvests. Biomass total lipid content and fatty acid composition were determined as described below.

### Microbial community analysis

DNA was extracted from 0.4 g (±5 mg) of microbial mat sample from each time point using the FastDNA Extraction Kit for Soil (MP Biomedicals, Santa Ana, CA, USA). The protocol of the manufacturer was slightly modified; the centrifugation time for all spin filter steps was increased to 10 min and additional purification steps were performed using 5.5 M guanidine thiocyanate.

The dual-index paired-end sequencing strategy was modified from the Metagenomic Sequencing Library Preparation document provided by Illumina. A two-step PCR method was used to generate 16S rRNA gene libraries. In the first step, PCR was used to amplify a region of DNA using region of interest-specific primers. 16S rRNA genes were amplified from the DNA extracts using amplicon primers S-D-Bact-0341-a-S-17 (5′-CCT ACG GGA GGC AGC AG-3′) and S-D-Bact-0785-a-A-21 (5′-GAC TAC HVG GGT ATC TAA TCC-3′) that were modified to include the Illumina forward (5′-TCG TCG GCA GCG TCA GAT GTG TAT AAG AGA CAG-3′) and reverse (5′-GTC TCG TGG GCT CGG AGA TGT GTA TAA GAG ACA G-3′) adapter sequences on their 5′ end. Primers Bact-0341-a-S-17 and S-D-Bact-0785-a-A-21 target the V3–V4 variable region (341–785 bp) of the 16S rRNA gene from *Bacteria* [50]. The homology between the bacterial 16S rRNA gene and the chloroplast 16S gene also leads to chloroplast 16S amplification [51]. All amplicon PCR reactions were performed in triplicate. PCR mixtures contained 0.1 µM of the forward primer, 0.1 µM of the reverse primer, 12.5 µL of 2× KAPA HiFi Hot Start Ready Mix (Kapa Biosystems, Wilmington, MA, USA) and 1 µL of template DNA (~1 ng µL^−1^), made up to 25 µL with nuclease-free water. PCR reaction conditions were as follows: initial denaturation at 95 °C for 3 min, followed by 25 cycles of 30 s at 95 °C, 45 s at 55 °C and 60 s at 72 °C, and a 5 min final elongation at 72 °C. Triplicate PCR reactions were pooled and purified using a 0.8× volume of AMPure XP magnetic beads (Beckman Coulter, Indianapolis, IN) as per the manufacturer’s instructions.

In the second step, PCR was used to attach dual indices and Illumina sequencing adapters to the amplicons from the first PCR reaction. These primers were complementary to the Illumina forward and reverse adapter sequences on the first PCR primers and included the P5 (forward) and P7 (reverse) adapter sequences to permit binding to the Illumina flow cell and an 8-base pair molecular barcode i5 (forward) or i7 (reverse). The general design of the sequencing primers were based on the Nextera XT i5 and i7 Index primer sequences. PCR mixtures contained 0.1 µM of the i5 index primer, 0.1 µM of the i7 index primer, 25 µL of 2× KAPA HiFi Hot Start Ready Mix (Kapa Biosystems, Wilmington, MA, USA) and 5 µL of template DNA, made up to 50 µL with nuclease-free water. PCR reaction conditions were: initial denaturation at 95 °C for 3 min, followed by 10 cycles of 30 s at 95 °C, 45 s at 55 °C and 60 s at 72 °C, and a 5 min final elongation at 72 °C. PCR reactions were purified using a 1.2× volume of AMPure XP magnetic beads (Beckman Coulter, Indianapolis, IN) as per the manufacturer’s instructions. Final amplicon library sizing was measured by performing region analysis on a Bioanalyzer 2100 (Agilent, Santa Clara, CA). DNA concentration was determined with a Qubit Fluorometer using a Quant-iT dsDNA HA Assay Kit (Life Technologies, Burlington, ON). Libraries were normalized and pooled for sequencing on the MiSeq Personal Sequencer (Illumina, San Diego, CA) using the 2 × 300 bp MiSeq Reagent Kit v3.

### Data analysis

The resulting amplicon sequences were analysed with MetaAmp (http://ebg.ucalgary.ca/metaamp), an in-house developed amplicon data analysis pipeline. In a first step, the paired-end raw reads from each sample went through the assembly (merging) process. The goal of the assembly is to convert a read pair into a longer single read containing one sequence and one set of quality scores. A pair is merged by aligning forward read sequence to the reverse-complement of the reverse reads sequence. In the overlap region where both reads cover the same regions, a single base and quality score are derived from the aligned pair of bases and quality scores for each position. MetaAmp assembled paired-end reads using “usearch -fastq_mergepairs”. The read pairs, which could not be aligned or whose overlap regions were shorter than 150 or the number of mismatches in the overlap region were greater than eight, were discarded. The assembled reads were further checked for the forward and reverse primers at the start and end of each read. The primers from the both ends were trimmed with mothur [52]. Those reads, which did not contain either forward and reverse primers or the number of mismatches in the primer region were greater than one, were discarded. The primer trimmed reads were subjected to quality filtering to remove low-quality reads and minimize the influence of sequencing errors. The quality filtering was done using “usearch -fastq_filter”. The reads were truncated to a fixed length of 350 bp and the total expected errors for all bases in each read after truncation greater than one were discarded. The reads with lengths shorter than the truncation length were also excluded from the further analysis. Finally, the unique sample ids were inserted into the header of the remaining high-quality reads and the reads from different samples were pooled together. The UPARSE pipeline [53] was used to identify chimeras and clustering the pooled high-quality reads into operational taxonomic units (OTUs). The OTU taxonomic assignment was achieved using the RDP classifier implemented in mothur with the SILAV training dataset as template. The UPARSE generated OTU table were converted into the OTU list file format which can be used by mothur. Mothur was then used to generate rarefaction data, the rank abundance data, the alpha-diversity (sobs, chao, ace, jackknife, Shannon, npshannon, simpson) and the beta-diversity [52]. The dissimilarities among different samples were calculated using the following measures: Jclass, Jest, thetayc and Bray–Curtis index. For each dissimilarity measure, a Newick-format tree was created to explore the hierarchical clustering of samples. In addition, two ordination methods, nonmetric multidimensional scaling (NMDS) and principal coordinate analysis (PCoA), were used to simplify the representation of dissimilarities by extracting a small number of axes, along which the samples show highest variability [54]. The identity of the top OTUs and chloroplast 16S rRNA genes were confirmed via comparison of the OTU reference sequences with GenBank sequences using the NCBI BLAST software package (http://blast.ncbi.nlm.nih.gov/Blast.cgi).

### Total lipid extraction and fatty acid identification

Approximately 65–100 mg of lyophilized sample was homogenized with a mortar and pestle and solvent extracted using a modified Bligh and Dyer method [55] as described previously [56]. Total lipid content was determined gravimetrically (w/dry weight %) from the total lipid extract (TLE). The TLE was subjected to an acid methanolysis for preparation of fatty acid methyl esters (FAMEs) from lipids including triacylglycerides, glycolipids, phospholipids, sterols esters and free fatty acids [57]. FAMEs were identified and quantified using gas chromatography mass spectrometry (GC/MS) on an Agilent GC–MS with DB5-MS capillary column (30 m × 0.25 mm I.D. × 0.25 µm film thickness) using a temperature programme of 50 °C (1 min), 20 °C min^−1^ to 130 °C, 4 °C min^−1^ to 160 °C, 8 °C min^−1^ to 300 °C (5 min) [56]. Identification was made based on the retention time and mass spectra of known reference standards (Bacterial Acid Methyl Esters Mix, Matreya Inc. and 37 FAME mix Supelco). FAMEs are named as follows; number of carbons:number of double bonds. Iso- and anteiso- are denoted by i or a, respectively. Br indicates a branch at an unknown location followed by the total number of carbons. Cy indicates cyclopropyl (see Additional file 9: Table S7).

## Additional files

**Additional file 1: Table S1.** Summary of microbial community composition detected in Deer Lake (DL-M) bioreactors on day 58, 85, 98 and 128 using 16S rRNA gene sequencing. Day 0 microbial mat sample is taken from the initial inoculation. Numbers indicate proportion of reads with a >1% average relative abundance in an OTU compared to all OTUs listed on a per sample basis. Closest cultured relative as determined by BLAST search is shown.

**Additional file 2: Table S2.** Summary of microbial community composition detected in Goodenough Lake (GEL-M) bioreactors on day 58, 85, 98 and 128 using 16S rRNA gene sequencing. Day 0 microbial mat sample is taken from the initial inoculation. Numbers indicate proportion of reads with a >1% average relative abundance in an OTU compared to all OTUs listed on a per sample basis. Closest cultured relative as determined by BLAST search is shown.

**Additional file 3: Table S3.** Summary of microbial community composition detected in Last Chance Lake (LCL-M) bioreactors on day 58, 85, 98 and 128 using 16S rRNA gene sequencing. Day 0 microbial mat sample is taken from the initial inoculation. Numbers indicate proportion of reads with a >1% average relative abundance in an OTU compared to all OTUs listed on a per sample basis. Closest cultured relative as determined by BLAST search is shown.

**Additional file 4: Table S4.** Summary of microbial community composition detected in Probe Lake (PL-M) bioreactors on day 58, 85, 98 and 128 using 16S rRNA gene sequencing. Day 0 microbial mat sample is taken from the initial inoculation. Numbers indicate proportion of reads with a >1% average relative abundance in an OTU compared to all OTUs listed on a per sample basis. Closest cultured relative as determined by BLAST search is shown.

**Additional file 5: Figure S1.** Nonmetric multidimensional scaling plot of soda lake bioreactor bacterial communities based on Bray–Curtis similarity showing separation of bioreactors based on time. Sample points formatted by soda lake Deer Lake (DL-M) (∆), Probe Lake (PL-M) (▼), Lake Chance Lake (LCL-M) (□) and Good Enough Lake (GEL-M) (○) and sampling timepoint (yellow, d 0; black, d 58; green, d 85; red, d 98; white, d 128).

**Additional file 6: Table S5.** Most abundant OTUs (>1% average relative abundance) and closest cultured relatives determined by BLAST search for the wavelength bioreactors.

**Additional file 7: Figure S2.** Nonmetric multidimensional scaling plot of biofilm microbial communities based on Bray–Curtis showing separation of microbial communities based on wavelength of light. Symbols indicate wavelength of light red (▼), white (○) and blue (□).

**Additional file 8: Table S6.** Most abundant OTUs (>1% average relative abundance) and closest cultured relatives determined by BLAST search for the high productivity (HP) bioreactor.

**Additional file 9: Table S7.** Fatty acid profile of the *Phormidium kuetzingianum* dominated biomass from the red photobioreactor at pH 9.0 and 0.5 M carbonates (day 20).

**Additional file 10: Table S8.** Characteristics of the four soda lakes.

**Additional file 11: Figure S3.** Design of the flat panel photobioreactor system showing (a) a schematic of the photobioreactor design and media flow pattern (b) a photo of an individual photobioreactor, and (c) a photo of the photobioreactor system in operation.

## Abbreviations

BECCS: bioenergy with carbon capture and storage
IPCC: International Panel on Climate Change
CO_2_: carbon dioxide
LCL-M: Last Chance Lake mat
PL-M: Probe Lake mat
DL-M: Deer Lake mat
GEL-M: Goodenough Lake mat
RAB: rotating algal biofilm
MUFA: monounsaturated fatty acids
PUFA: polyunsaturated fatty acids
EDTA: ethylenediaminetetraacetic acid
PAR: photosynthetically active radiation
OTU: operational taxonomic unit
NMDS: nonmetric multidimensional scaling
PCoA: principal coordinate analysis
TLE: total lipid extract
FAMEs: fatty acid methyl esters
GC/MS: gas chromatography mass spectrometry
