# Supplementary material for: Robust, high-productivity phototrophic carbon capture at high pH and alkalinity using natural microbial communities
Source: Biotechnol Biofuels. 2017 Mar 29;10:84. doi: 10.1186/s13068-017-0769-1 (PMC5372337; doi:10.1186/s13068-017-0769-1)
Supplement: Supplementary file 1 — Additional file 1: Table S1. Summary of microbial community composition detected in Deer Lake (DL-M) bioreactors on day 58, 85, 98 and 128 using 16S rRNA gene sequencing. Day 0 microbial mat sample is taken from the initial inoculation. Numbers indicate proportion of reads with a >1% average relative abundance in an OTU compared to all OTUs listed on a per sample basis. Closest cultured relative as determined by BLAST search is shown. [file 13068_2017_769_MOESM1_ESM.pdf]

**Table S1**. Summary of microbial community composition detected in Deer Lake (DL-M) bioreactors on day 58, 85, 98 and 128 using 16S rRNA gene sequencing. Day 0 microbial mat sample is taken from the initial inoculation. Numbers indicate proportion of reads with a > 1% average relative abundance in an OTU compared to all OTUs listed on a per sample basis. Closest cultured relative as determined by BLAST search is shown.

| OTU Number | % total DL-M OTUs | Relative % abundance in duplicate DL-M bioreactors at time point (day) | | | | | | | | | Phylum | Genus | Accession No. | % Identity |
|---|---|---|---|---|---|---|---|---|---|---|---|---|---|---|
| | | 0 | 58 | | 85 | | 98 | | 128 | | | | | |
| | | | BR1 | BR2 | BR1 | BR2 | BR1 | BR2 | BR1 | BR2 | | | | |
| 2 | 18.43 | 1.01 | 3.02 | 3.57 | 16.61 | 24.10 | 20.98 | 31.59 | 40.82 | 33.36 | *Proteobacteria* | *Wenzhouxiangella* | CP012154.1 | 98 |
| 1 | 17.15 | 2.04 | 45.11 | 39.81 | 37.48 | 26.76 | 0.40 | 0.80 | 0.06 | 0.11 | *Bacillariophyta* | *Dickieia* | FJ002229.1 | 99 |
| 5 | 8.37 | 2.42 | 3.11 | 3.94 | 5.82 | 4.88 | 14.34 | 11.88 | 14.19 | 22.39 | *Proteobacteria* | *Rhodobaca* | EU908048.1 | 100 |
| 3 | 6.37 | 0.02 | 2.07 | 9.71 | 14.34 | 19.12 | 10.48 | 0.48 | 2.04 | 0.56 | *Cyanobacteria* | *Phormidium* | JN166666.1 | 100 |
| 7 | 5.94 | 0.68 | 2.13 | 3.21 | 4.59 | 2.60 | 13.08 | 13.27 | 3.85 | 3.21 | *Bacteroidetes* | *Lewinella* | NR_115013.1 | 89 |
| 6 | 4.35 | 1.18 | 0.41 | 0.68 | 1.98 | 4.15 | 10.04 | 9.69 | 3.85 | 4.86 | *Proteobacteria* | *Chelatococcus* | NR_025428.1 | 98 |
| 4 | 4.16 | 0.51 | 28.18 | 7.49 | 4.64 | 2.30 | 0.40 | 0.14 | 0.09 | 0.09 | *Bacillariophyta* | *Nitzschia* | FJ002224.1 | 99 |
| 245 | 3.01 | 0.22 | 4.19 | 3.90 | 1.14 | 1.81 | 3.50 | 5.70 | 2.41 | 4.39 | *Bacteroidetes* | *Lewinella* | KF228160.1 | 89 |
| 8 | 2.27 | 1.30 | 0.17 | 0.71 | 1.48 | 1.77 | 4.03 | 3.48 | 6.05 | 3.23 | *Proteobacteria* | *Rhodobaculum* | KM077018.1 | 99 |
| 40 | 1.31 | 2.68 | 0.12 | 0.09 | 1.42 | 1.46 | 0.94 | 1.45 | 1.72 | 0.67 | *Proteobacteria* | *Halomonas* | GU113002.1 | 99 |
