# Supplementary material for: Robust, high-productivity phototrophic carbon capture at high pH and alkalinity using natural microbial communities
Source: Biotechnol Biofuels. 2017 Mar 29;10:84. doi: 10.1186/s13068-017-0769-1 (PMC5372337; doi:10.1186/s13068-017-0769-1)
Supplement: Supplementary file 2 — Additional file 2: Table S2. Summary of microbial community composition detected in Goodenough Lake (GEL-M) bioreactors on day 58, 85, 98 and 128 using 16S rRNA gene sequencing. Day 0 microbial mat sample is taken from the initial inoculation. Numbers indicate proportion of reads with a >1% average relative abundance in an OTU compared to all OTUs listed on a per sample basis. Closest cultured relative as determined by BLAST search is shown. [file 13068_2017_769_MOESM2_ESM.pdf]

Table S2. Summary of microbial community composition detected in Goodenough Lake (GEL-M) bioreactors on day 58, 85, 98 and 128 using 16S rRNA gene sequencing. Day 0 microbial mat sample is taken from the initial inoculation. Numbers indicate proportion of reads with a > 1% average relative abundance in an OTU compared to all OTUs listed on a per sample basis. Closest cultured relative as determined by BLAST search is shown.

OTU Number	% total GEL-M OTUs	Relative % abundance in duplicate GEL-M bioreactors at time point (day)									Phylum	Genus	Accession No.	% Identity
		0	58		85		98		128					
			BR1	BR2	BR1	BR2	BR1	BR2	BR1	BR2				
2	15.18	0.27	7.04	5.11	11.34	7.33	17.32	20.07	21.30	28.56	*Proteobacteria*	*Wenzhouxiangella*	CP012154.1	98
1	14.82	0.13	36.68	40.05	26.36	19.37	0.07	0.38	0.08	0.15	*Bacillariophyta*	*Dickieia*	FJ002229.1	99
5	10.97	0.84	2.99	3.82	3.71	3.88	10.49	13.17	20.70	21.35	*Proteobacteria*	*Rhodobaca*	EU908048.1	100
3	10.16	1.14	20.59	21.32	29.13	42.50	0.33	1.31	0.35	0.86	*Cyanobacteria*	*Phormidium*	JN166666.1	100
245	5.06	0.50	2.74	3.15	2.88	2.56	8.57	9.62	5.33	4.66	*Bacteroidetes*	*Lewinella*	KF228160.1	89
7	4.54	1.17	5.25	5.25	3.90	3.91	6.47	8.15	2.40	2.08	*Bacteroidetes*	*Lewinella*	NR_115013.1	89
8	4.40	0.97	0.40	0.35	1.05	0.97	4.69	4.25	5.76	12.53	*Proteobacteria*	*Rhodobaculum*	KM077018.1	99
6	3.93	0.37	2.64	1.22	3.02	1.66	5.87	3.19	7.67	4.52	*Proteobacteria*	*Chelatococcus*	NR_025428.1	98
10	2.86	0.00	2.94	3.05	0.57	0.92	6.26	4.05	1.46	0.58	*Verrucomicrobia*	*Coraliomargarita*	NR_074901.1	90
9	2.69	0.00	0.01	0.00	0.55	0.40	8.08	5.13	1.85	2.70	*Bacteroidetes*	*Fabibacter*	NR_137379.1	91
11	1.74	0.00	0.26	0.15	0.50	1.37	1.58	3.40	4.64	2.57	*Deinococcus-Thermus*	*Truepera*	NR_074381.1	88
13	1.32	0.00	0.00	0.00	0.07	0.00	5.19	0.00	2.04	0.00	*Firmicutes*	*Alkalibacterium*	NR_112659.1	99
28	1.16	0.97	0.35	0.22	0.28	0.38	1.54	1.60	2.33	1.77	*Proteobacteria*	*Wenzhouxiangella*	CP012154.1	96
12	1.08	0.50	0.57	0.34	0.81	0.49	1.42	1.54	1.96	1.37	*Proteobacteria*	*Thioalkalivibrio*	NR_074692.1	95
