# Supplementary material for: Robust, high-productivity phototrophic carbon capture at high pH and alkalinity using natural microbial communities
Source: Biotechnol Biofuels. 2017 Mar 29;10:84. doi: 10.1186/s13068-017-0769-1 (PMC5372337; doi:10.1186/s13068-017-0769-1)
Supplement: Supplementary file 3 — Additional file 3: Table S3. Summary of microbial community composition detected in Last Chance Lake (LCL-M) bioreactors on day 58, 85, 98 and 128 using 16S rRNA gene sequencing. Day 0 microbial mat sample is taken from the initial inoculation. Numbers indicate proportion of reads with a >1% average relative abundance in an OTU compared to all OTUs listed on a per sample basis. Closest cultured relative as determined by BLAST search is shown. [file 13068_2017_769_MOESM3_ESM.pdf]

Table S3. Summary of microbial community composition detected in Last Chance Lake (LCL-M) bioreactors on day 58, 85, 98 and 128 using 16S rRNA gene sequencing. Day 0 microbial mat sample is taken from the initial inoculation. Numbers indicate proportion of reads with a > 1% average relative abundance in an OTU compared to all OTUs listed on a per sample basis. Closest cultured relative as determined by BLAST search is shown.

OTU Number	% total LCL-M OTUs	Relative % abundance in duplicate LCL-M bioreactors at time point (day)									Phylum	Genus	Accession No.	% Identity
		0	58		85		98		128					
			BR1	BR2	BR1	BR2	BR1	BR2	BR1	BR2				
2	14.13	0.68	1.64	4.07	12.12	10.90	25.92	32.31	29.43	22.96	*Proteobacteria*	*Wenzhouxiangella*	CP012154.1	98
5	13.52	21.58	3.98	5.27	4.59	3.47	18.16	14.98	24.42	28.39	*Proteobacteria*	*Rhodobaca*	EU908048.1	100
4	11.11	1.52	8.00	42.92	31.17	12.48	3.02	0.83	0.15	0.71	*Bacillariophyta*	*Nitzschia*	FJ002224.1	99
1	10.69	0.64	41.05	6.86	12.72	6.73	0.37	0.05	0.02	0.13	*Bacillariophyta*	*Dickieia*	FJ002229.1	99
3	7.95	0.10	9.27	16.99	12.68	45.39	7.50	0.93	0.15	0.16	*Cyanobacteria*	*Phormidium*	JN166666.1	100
8	3.77	0.50	0.31	1.79	1.56	1.78	6.69	6.87	11.45	6.53	*Proteobacteria*	*Rhodobaculum*	KM077018.1	99
245	3.15	0.68	4.14	5.29	6.93	2.36	2.79	2.40	0.70	1.14	*Bacteroidetes*	*Lewinella*	KF228160.1	89
6	2.94	2.32	0.52	0.63	1.01	0.71	4.20	3.17	9.53	7.16	*Proteobacteria*	*Chelatococcus*	NR_025428.1	98
9	1.97	0.01	0.00	0.00	0.00	2.99	0.70	16.74	0.39	1.19	*Bacteroidetes*	*Fabibacter*	NR_137379.1	91
128	1.88	0.82	0.35	0.29	0.36	0.37	3.77	2.13	3.13	5.69	*Proteobacteria*	*Paracoccus*	NR_116733.1	99
22	1.49	3.60	5.00	0.08	0.13	0.30	0.11	0.02	0.00	0.00	*Cyanobacteria*	*Halomicronema*	GU220365.1	94
18	1.47	0.69	2.80	0.84	1.02	0.10	1.41	0.31	0.21	3.31	*Proteobacteria*	*Glycocaulis*	KU561631.1	96
13	1.20	0.03	0.02	0.00	0.05	0.34	6.38	0.51	0.27	0.15	*Firmicutes*	*Alkalibacterium*	NR_112659.1	99
