# Supplementary material for: Robust, high-productivity phototrophic carbon capture at high pH and alkalinity using natural microbial communities
Source: Biotechnol Biofuels. 2017 Mar 29;10:84. doi: 10.1186/s13068-017-0769-1 (PMC5372337; doi:10.1186/s13068-017-0769-1)
Supplement: Supplementary file 4 — Additional file 4: Table S4. Summary of microbial community composition detected in Probe Lake (PL-M) bioreactors on day 58, 85, 98 and 128 using 16S rRNA gene sequencing. Day 0 microbial mat sample is taken from the initial inoculation. Numbers indicate proportion of reads with a >1% average relative abundance in an OTU compared to all OTUs listed on a per sample basis. Closest cultured relative as determined by BLAST search is shown. [file 13068_2017_769_MOESM4_ESM.pdf]

Table S4. Summary of microbial community composition detected in Probe Lake (PL-M) bioreactors on day 58, 85, 98 and 128 using 16S rRNA gene sequencing. Day 0 microbial mat sample is taken from the initial inoculation. Numbers indicate proportion of reads with a > 1% average relative abundance in an OTU compared to all OTUs listed on a per sample basis. Closest cultured relative as determined by BLAST search is shown.

OTU Number	% total PL-M OTUs	Relative % abundance in duplicate PL-M bioreactors at time point (day)									Phylum	Genus	Accession No.	% Identity
		0	58		85		98		128					
			BR1	BR2	BR1	BR2	BR1	BR2	BR1	BR2				
2	16.03	2.40	4.97	5.32	9.93	6.94	21.46	20.89	34.43	31.18	*Proteobacteria*	*Wenzhouxiangella*	CP012154.1	98
1	14.12	1.12	23.37	31.48	33.21	28.57	0.25	0.08	0.16	0.31	*Bacillariophyta*	*Dickieia*	FJ002229.1	99
3	10.76	1.05	24.87	11.68	20.60	33.64	0.34	0.38	0.16	0.59	*Cyanobacteria*	*Phormidium*	JN166666.1	100
5	8.63	6.69	2.78	4.98	3.76	2.75	11.67	18.81	9.88	18.73	*Proteobacteria*	*Rhodobaca*	EU908048.1	100
8	6.41	2.04	0.65	1.29	2.59	1.80	9.13	7.43	16.07	15.58	*Proteobacteria*	*Rhodobaculum*	KM077018.1	99
4	5.62	1.65	13.79	25.22	4.73	7.24	0.22	0.13	0.10	0.53	*Bacillariophyta*	*Nitzschia*	FJ002224.1	99
7	4.01	0.24	3.41	2.64	4.17	3.64	8.71	5.52	2.64	1.33	*Bacteroidetes*	*Lewinella*	NR_115013.1	89
245	3.50	0.37	4.23	1.41	3.80	3.19	7.16	5.89	1.34	0.78	*Bacteroidetes*	*Lewinella*	KF228160.1	89
6	3.18	1.44	1.26	0.71	1.39	1.00	4.02	4.06	7.65	6.16	*Proteobacteria*	*Chelatococcus*	NR_025428.1	98
19	1.77	12.88	0.08	0.06	0.17	0.10	3.22	1.58	1.56	0.93	*Cyanobacteria*	*Cyanobacteria*	NR_102450.1	98
12	1.18	0.51	0.68	0.44	0.70	0.50	2.48	2.36	1.12	1.19	*Proteobacteria*	*Thioalkalivibrio*	NR_074692.1	95
11	1.06	0.01	0.05	0.05	0.65	0.99	1.85	2.51	1.11	1.90	*Deinococcus-Thermus*	*Truepera*	NR_074381.1	88
18	1.04	0.12	0.12	0.06	0.24	0.42	1.04	2.39	1.54	4.42	*Proteobacteria*	*Glycocaulis*	KU561631.1	96
