# Supplementary material for: Robust, high-productivity phototrophic carbon capture at high pH and alkalinity using natural microbial communities
Source: Biotechnol Biofuels. 2017 Mar 29;10:84. doi: 10.1186/s13068-017-0769-1 (PMC5372337; doi:10.1186/s13068-017-0769-1)
Supplement: Supplementary file 5 — Additional file 5: Figure S1. Nonmetric multidimensional scaling plot of soda lake bioreactor bacterial communities based on Bray–Curtis similarity showing separation of bioreactors based on time. Sample points formatted by soda lake Deer Lake (DL-M) (∆), Probe Lake (PL-M) (▼), Lake Chance Lake (LCL-M) (□) and Good Enough Lake (GEL-M) (○) and sampling timepoint (yellow, d 0; black, d 58; green, d 85; red, d 98; white, d 128). [file 13068_2017_769_MOESM5_ESM.pdf]

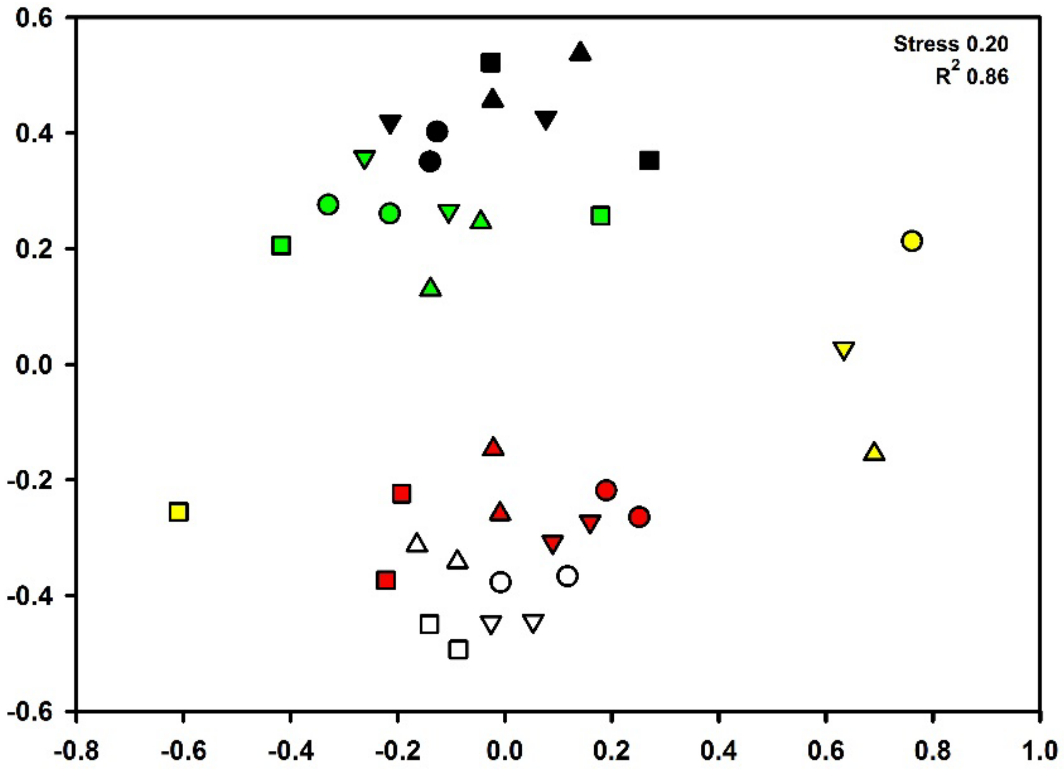

Figure S1. Nonmetric multidimensional scaling plot of soda lake bioreactor bacterial communities based on Bray-Curtis similarity showing separation of bioreactors based on time. Sample points formatted by soda lake Deer Lake (DL-M) (Δ), Probe Lake (PL-M) (▼), Lake Chance Lake (LCL-M) (□) and Good Enough Lake (GEL-M) (○) and sampling timepoint (yellow, d 0; black, d 58; green, d 85; red, d 98; white, d 128).
