# Supplementary material for: Robust, high-productivity phototrophic carbon capture at high pH and alkalinity using natural microbial communities
Source: Biotechnol Biofuels. 2017 Mar 29;10:84. doi: 10.1186/s13068-017-0769-1 (PMC5372337; doi:10.1186/s13068-017-0769-1)
Supplement: Supplementary file 6 — Additional file 6: Table S5. Most abundant OTUs (>1% average relative abundance) and closest cultured relatives determined by BLAST search for the wavelength bioreactors. [file 13068_2017_769_MOESM6_ESM.pdf]

**Table S5.** Most abundant OTUs (>1% average relative abundance) and closest cultured relatives determined by BLAST search for the wavelength bioreactors.

| OTU | Average Relative Abundance (%) | | | Phylum | Genus | Accession No. | % Identity |
|---|---|---|---|---|---|---|---|
| | Red | White | Blue | | | | |
| 1 | <1 | 32.52 | 87.82 | *Bacillariophyta* | *Nitzschia* | FJ002224.1 | 99 |
| 2 | 63.07 | 34.49 | <1 | *Cyanobacteria* | *Phormidium* | JN166666.1 | 100 |
| 3 | 8.07 | 7.17 | 1.37 | *Proteobacteria* | *Wenzhouxiangella* | CP012154.1 | 100 |
| 4 | 2.16 | 2.00 | <1 | *Proteobacteria* | *Chelatococcus* | NR_025428.1 | 98 |
| 5 | 2.08 | 1.77 | <1 | *Bacteroidetes* | *Lewinella* | NR_115013.1 | 89 |
| 6 | <1 | 1.90 | <1 | *Cyanobacteria* | *Cyanobacterium* | NR_102450.1 | 98 |
| 8 | 3.06 | 2.71 | 1.13 | *Proteobacteria* | *Rhodobaca* | EU908048.1 | 100 |
| 9 | 2.02 | 1.05 | <1 | *Cyanobacteria* | *Phormidium* | HM446280.1 | 94 |
| 11 | 1.86 | <1 | <1 | *Proteobacteria* | *Salinispirillum* | NR_134169.1 | 94 |
| 12 | 1.26 | <1 | <1 | *Proteobacteria* | *Marinobacter* | KC534446.1 | 96 |
| 162 | 1.75 | 2.31 | <1 | *Bacteroidetes* | *Lewinella* | KF228160.1 | 89 |
