# Supplementary material for: Robust, high-productivity phototrophic carbon capture at high pH and alkalinity using natural microbial communities
Source: Biotechnol Biofuels. 2017 Mar 29;10:84. doi: 10.1186/s13068-017-0769-1 (PMC5372337; doi:10.1186/s13068-017-0769-1)
Supplement: Supplementary file 7 — Additional file 7: Figure S2. Nonmetric multidimensional scaling plot of biofilm microbial communities based on Bray–Curtis showing separation of microbial communities based on wavelength of light. Symbols indicate wavelength of light red (▼), white (○) and blue (□). [file 13068_2017_769_MOESM7_ESM.pdf]

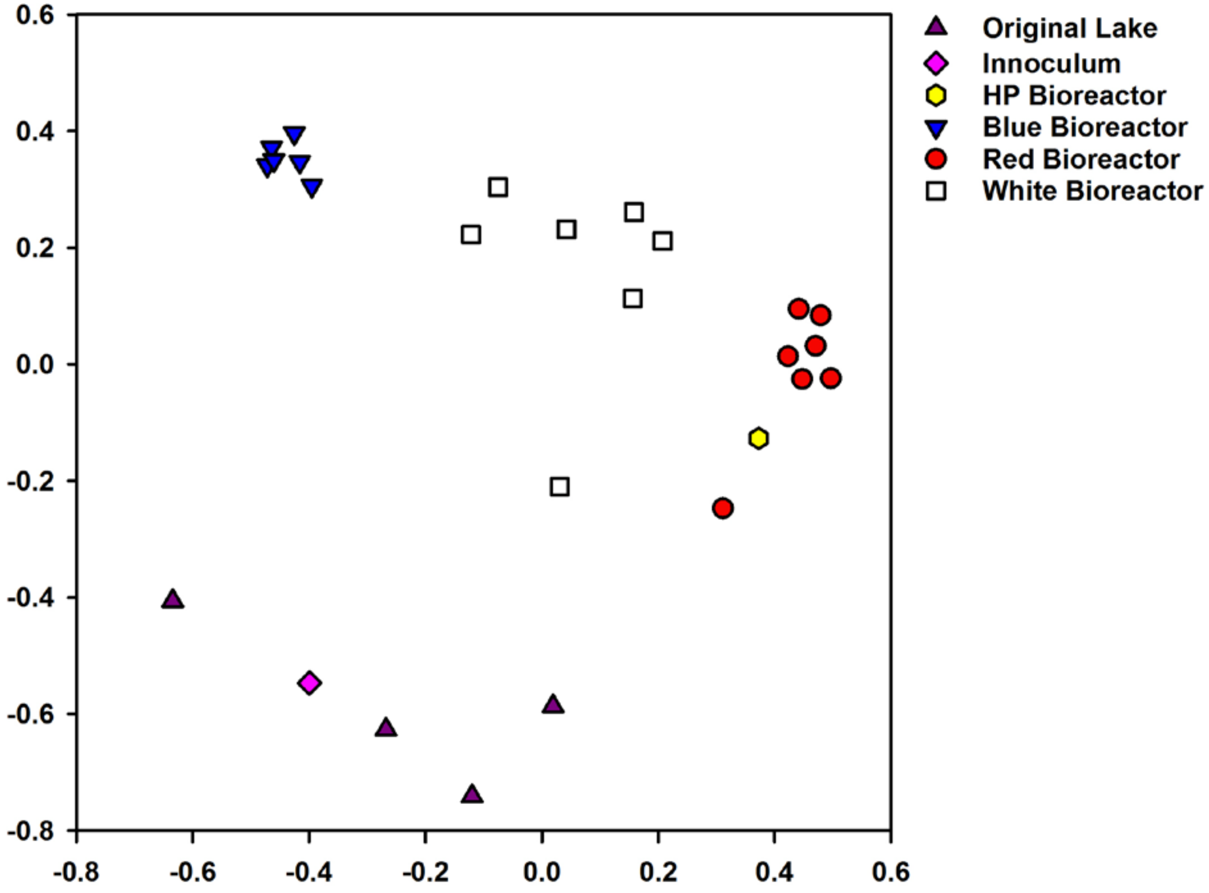

Figure S2. Nonmetric multidimensional scaling plot of biofilm microbial communities based on Bray-Curtis showing separation of microbial communities based on wavelength of light. Symbols indicate wavelength of light red (▼), white (○) and blue (□).
