# Supplementary material for: Robust, high-productivity phototrophic carbon capture at high pH and alkalinity using natural microbial communities
Source: Biotechnol Biofuels. 2017 Mar 29;10:84. doi: 10.1186/s13068-017-0769-1 (PMC5372337; doi:10.1186/s13068-017-0769-1)
Supplement: Supplementary file 8 — Additional file 8: Table S6. Most abundant OTUs (>1% average relative abundance) and closest cultured relatives determined by BLAST search for the high productivity (HP) bioreactor. [file 13068_2017_769_MOESM8_ESM.pdf]

Table S6. Most abundant OTUs (>1% average relative abundance) and closest cultured relatives determined by BLAST search for the high productivity (HP) bioreactor.

OTU	Average Relative Abundance (%)	Phylum	Genus	Accession No.	% Identity
2	53.76	*Cyanobacteria*	*Phormidium*	JN166666.1	100
3	10.56	*Proteobacteria*	*Chelatococcus*	NR_025428.1	98
1	4.82	*Bacillariophyta*	*Nitzschia*	FJ002224.1	99
4	4.61	*Proteobacteria*	*Wenzhouxiangella*	CP012154.1	100
6	2.99	*Proteobacteria*	*Rhodobaca*	EU908048.1	100
8	2.88	*Bacteroidetes*	*Owenweeksia*	NR_074100.1	90
22	1.97	*Bacteroidetes*	*Brumimicrobium*	LT158299.1	92
9	1.55	*Verrucomicrobia*	*Fucophilus*	NR_112167.1	96
11	1.46	*Proteobacteria*	*Salinispirillum*	NR_134169.1	94
7	1.39	*Bacteroidetes*	*Lewinella*	KF228160.1	90
20	1.33	*Bacteroidetes*	*Psychroflexus*	KJ782429.2	96
472	1.21	*Bacteroidetes*	*Lewinella*	KF228160.1	89
