# Supplementary material for: Robust, high-productivity phototrophic carbon capture at high pH and alkalinity using natural microbial communities
Source: Biotechnol Biofuels. 2017 Mar 29;10:84. doi: 10.1186/s13068-017-0769-1 (PMC5372337; doi:10.1186/s13068-017-0769-1)
Supplement: Supplementary file 9 — Additional file 9: Table S7. Fatty acid profile of the Phormidium kuetzingianum dominated biomass from the red photobioreactor at pH 9.0 and 0.5 M carbonates (day 20). [file 13068_2017_769_MOESM9_ESM.pdf]

Table S7. Fatty acid profile of the *Phormidium kuetzingianum* dominated biomass from the red photobioreactor at pH 9.0 and 0.5 M carbonates (day 20).

Fatty Acid	mol % total fatty acid
br13:0	0.7
14:0	3.0
15:0	0.5
i15:0	0.7
15:1	0.4
16:0	26.7
i16:0	0.4
16:1	36.7
16:2	10.3
16:4	0.5
17:0	0.2
cy17:0	0.6
i17:0	0.2
18:0	0.6
18:1	5.8
18:2	0.9
18:3	1.6
cy19:0	0.6
20:4	1.3
20:5	8.0
22:6	0.3
