# Supplementary material for: Robust, high-productivity phototrophic carbon capture at high pH and alkalinity using natural microbial communities
Source: Biotechnol Biofuels. 2017 Mar 29;10:84. doi: 10.1186/s13068-017-0769-1 (PMC5372337; doi:10.1186/s13068-017-0769-1)
Supplement: Supplementary file 10 — Additional file 10: Table S8. Characteristics of the four soda lakes. [file 13068_2017_769_MOESM10_ESM.pdf]

Table S8. Characteristics of the four soda lakes.

**Sample name**	**Description**	**Location**	**Temp. (^{o}C)**	**pH**	**GPS Coordinates**
DL-M	Deer Lake	Cariboo Plateau, Canada	19.6	10.3	51°21'15.42"N 121°14'43.44"W
GEL-M	Goodenough Lake	Cariboo Plateau, Canada	24.9	10.3	51°19'47.64"N 121°38'28.90"W
LCL-M	Last Chance Lake	Cariboo Plateau, Canada	25.2	10.2	51°19'39.3" N 121°37'59.3"W
PL-M	Probe Lake	Cariboo Plateau, Canada	15.5	10.3	51°27'2.70"N 121°23'15.30"W
