# Supplementary material for: Robust, high-productivity phototrophic carbon capture at high pH and alkalinity using natural microbial communities
Source: Biotechnol Biofuels. 2017 Mar 29;10:84. doi: 10.1186/s13068-017-0769-1 (PMC5372337; doi:10.1186/s13068-017-0769-1)
Supplement: Supplementary file 11 — Additional file 11: Figure S3. Design of the flat panel photobioreactor system showing (a) a schematic of the photobioreactor design and media flow pattern (b) a photo of an individual photobioreactor, and (c) a photo of the photobioreactor system in operation. [file 13068_2017_769_MOESM11_ESM.pdf]

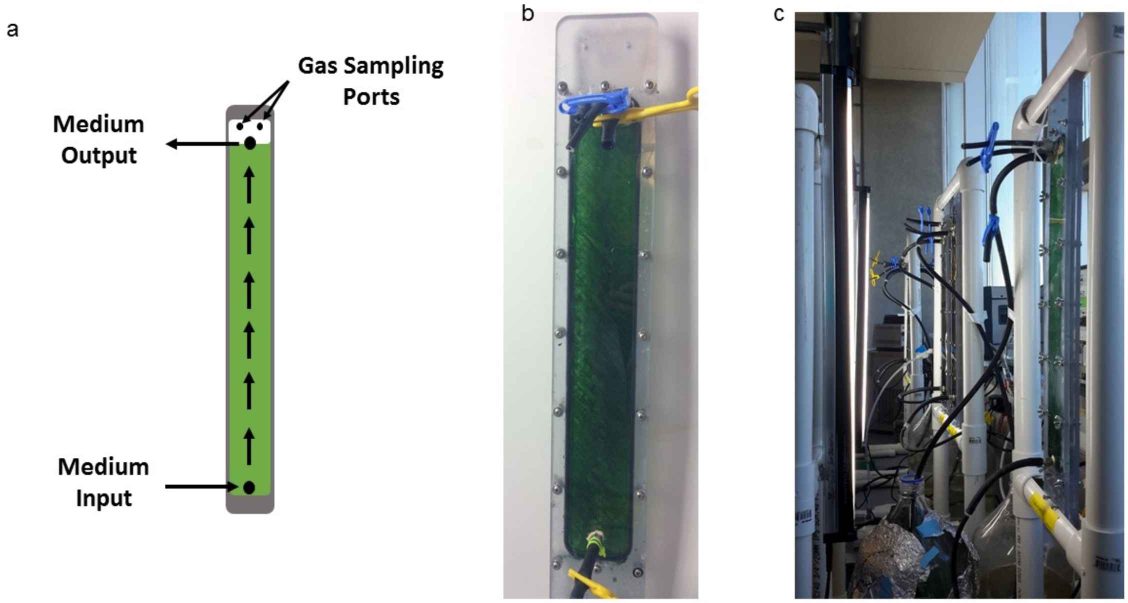

Figure S3. Design of the flat panel photobioreactor system showing (a) a schematic of the photobioreactor design and media flow pattern, (b) a photo of an individual photobioreactor, and (c) a photo of the photobioreactor system in operation.
